# More than Hormones? Metabolic Effects of Twelve-Week Probiotic Supplementation in Women with Polycystic Ovary Syndrome: A Secondary Analysis of a Randomized Double-Blind Placebo-Controlled Study

**DOI:** 10.3390/nu18183090

**Published:** 2026-09-21

**Authors:** Amalia Gorzko, Jolanta Nawrocka-Rutkowska, Małgorzata Szczuko, Hubert Pawłowski, Andrzej Starczewski, Iwona Szydłowska

**Affiliations:** 1 Department of Gynecology, Endocrinology and Gynecological Oncology, Pomeranian Medical University in Szczecin, 71-252 Szczecin, Poland; 2 Department of Bromatology and Nutritional Diagnostics, Pomeranian Medical University in Szczecin, 71-252 Szczecin, Poland; 3 Klinik für Anästhesiologie und Intensivmedizin, Charité–Universitätsmedizin Berlin, 10117 Berlin, Germany

**Keywords:** polycystic ovary syndrome, probiotics, glucose metabolism, insulin resistance, lipid profile, gut microbiota

## Abstract

**Background:** Polycystic ovary syndrome (PCOS) is frequently associated with insulin resistance and dyslipidemia. Evidence regarding the metabolic effects of probiotics in PCOS remains inconsistent. This secondary analysis evaluated whether multispecies probiotic supplementation influenced glucose homeostasis, insulin resistance, and the lipid profile in women with PCOS. **Methods:** Data were derived from a 12-week randomized, double-blind, placebo-controlled study in which 50 women with PCOS were assigned to receive either a nine-strain probiotic (1.0 × 10^9^ CFU/day) or placebo. Fasting and 120 min glucose and insulin concentrations, HOMA-IR, total cholesterol, HDL cholesterol, LDL cholesterol, and triglycerides were assessed at baseline and follow-up. Treatment effects were evaluated using analysis of covariance adjusted for baseline values. The main analysis was based on participants with available follow-up data, and an intention-to-treat sensitivity analysis including all 50 randomized participants was performed using multiple imputation for missing follow-up measurements. **Results:** Forty-three participants were included in the complete-case analysis (probiotic, *n* = 19; placebo, *n* = 24); seven did not complete follow-up (six in the probiotic group, one in the placebo group). No statistically significant between-group differences were detected for glucose homeostasis, HOMA-IR, or lipid profile. The intention-to-treat sensitivity analysis yielded findings consistent with the complete-case analysis. **Conclusions:** Twelve weeks of multispecies probiotic supplementation did not result in statistically significant improvements in glucose metabolism, insulin resistance, or lipid profile compared with placebo. Given the relatively small sample size and limited precision of some estimates, clinically relevant effects cannot be excluded. Larger randomized controlled trials are warranted.

## 1. Introduction

Polycystic ovary syndrome affects approximately 10–13% of women of reproductive age, making it one of the most common endocrine disorders worldwide [1]. Although several diagnostic frameworks have been proposed, the Rotterdam criteria remain the most widely applied approach for PCOS diagnosis in both clinical practice and scientific research. They require the presence of at least two of the following three features, after excluding other disorders with similar clinical manifestations: oligo- or anovulation, clinical and/or biochemical hyperandrogenism, and polycystic ovarian morphology on ultrasonography [2].

Over the past decade, the perception of PCOS has shifted from a predominantly reproductive disorder to a complex metabolic–endocrine condition. Beyond reproductive dysfunction, women with PCOS frequently present with insulin resistance, compensatory hyperinsulinemia, dyslipidemia, and an elevated risk of metabolic syndrome, type 2 diabetes mellitus, and cardiovascular disease [3]. The metabolic abnormalities observed in PCOS result from a complex interplay between genetic predisposition, hormonal disturbances, adipose tissue dysfunction, and chronic low-grade inflammation [4,5]. Insulin resistance represents a key metabolic hallmark of PCOS and may occur independently of obesity, although excess adiposity can further exacerbate impaired insulin signaling [6]. Compensatory hyperinsulinemia promotes ovarian androgen excess and contributes to the maintenance of hormonal imbalance characteristic of PCOS [7]. This paradigm shift has prompted a global consensus process that proposed the term polyendocrine metabolic ovarian syndrome (PMOS), emphasizing the multisystemic endocrine and metabolic nature of the disorder [8]. Nevertheless, PCOS remains the established terminology in current clinical practice and scientific literature.

Among the mechanisms potentially contributing to metabolic dysfunction in PCOS, increasing attention has been directed toward alterations in the gut microbiome. The gut microbiota has emerged as an important regulator of host metabolism, immune function, and endocrine signaling [9]. Gut dysbiosis, characterized by alterations in microbial composition and function, has been increasingly recognized as a potential contributor to the metabolic abnormalities observed in women with PCOS [7,9]. Through modulation of host metabolism, immune responses, and endocrine signaling, alterations in gut microbial communities may represent an important link between metabolic dysfunction and the clinical manifestations of the syndrome [10].

Several mechanisms may explain the association between gut microbiota alterations and PCOS pathophysiology. Microbial metabolites, including short-chain fatty acids (SCFAs), may influence glucose metabolism, energy homeostasis, and inflammatory responses, whereas impaired intestinal barrier integrity may promote lipopolysaccharide (LPS) translocation, metabolic endotoxemia, and activation of inflammatory pathways, thereby aggravating insulin resistance [11]. The gut microbiota may also influence steroid metabolism through the estrobolome; however, the clinical relevance of this pathway in PCOS remains incompletely understood [12].

These findings have led to growing interest in microbiota-modulating strategies. Probiotics, defined as live microorganisms that confer health benefits when administered in adequate amounts, and synbiotics, which combine probiotics with prebiotics, have emerged as potential therapeutic approaches in PCOS [13]. However, despite promising findings, current evidence remains inconclusive due to heterogeneity in strains, doses, and study protocols, warranting further clinical trials [14]. Moreover, most available evidence originates from Asian and Middle Eastern populations, whereas data from Central European populations, particularly from Polish women with PCOS, remain scarce.

In our previous analysis of the same randomized, placebo-controlled study, probiotic supplementation was associated with changes in hormonal parameters and BMI in women with PCOS [15]. Therefore, the aim of the present secondary analysis was to explore the potential effects of 12-week multispecies probiotic supplementation on glucose homeostasis and lipid profile in women with PCOS. We hypothesized that probiotic supplementation might be associated with changes in these metabolic parameters. The metabolic outcomes reported in the present analysis have not been previously published.

## 2. Materials and Methods

### 2.1. Study Group

A 12-week randomized, double-blind, placebo-controlled clinical study was conducted in women with PCOS. It was approved by the local Bioethical Committee of Pomeranian Medical University in Szczecin (Approval Code: KB-0012/169/19) and complied with the principles of the Declaration of Helsinki. The study was retrospectively registered with ClinicalTrials.gov (NCT06761625). The present manuscript reports a secondary analysis of the randomized clinical study. The metabolic parameters analyzed in this study were planned as part of the original study procedures and were prospectively assessed at baseline and after the 12-week intervention. These outcomes were not specified as primary or secondary endpoints in the ClinicalTrials.gov registration. All participants provided written informed consent before enrollment.

The study included 50 women with PCOS diagnosed according to the Rotterdam ESHRE criteria, as described in the Introduction. Polycystic ovarian morphology was assessed by transvaginal ultrasonography and defined as ≥20 follicles in at least one ovary and/or an ovarian volume ≥ 10 mL. Participants underwent transvaginal ultrasonography using an Alpinion X-CUBE 70 system with an 11 MHz probe. Women using hormonal therapy within 3 months or medications potentially affecting study outcomes within 1 month prior to enrollment were excluded. No concomitant pharmacological treatment targeting glucose metabolism, insulin resistance, or lipid metabolism was used during the intervention. Additional exclusion criteria included type 1 or type 2 diabetes mellitus, cardiovascular diseases, liver diseases, hypertension, and thyroid disorders. Other causes of hyperandrogenism and ovulatory dysfunction, including congenital adrenal hyperplasia and androgen-secreting ovarian tumors, were excluded as part of the diagnostic assessment before PCOS diagnosis. Anthropometric measurements were assessed at baseline and after 12 weeks, and BMI was calculated as weight (kg)/height^2^ (m^2^) [16]. Insulin resistance was estimated using the homeostatic model assessment of insulin resistance (HOMA-IR), calculated as fasting insulin (mIU/L) × fasting glucose (mmol/L)/22.5 [17], and analyzed as a continuous variable.

**Figure 1:**
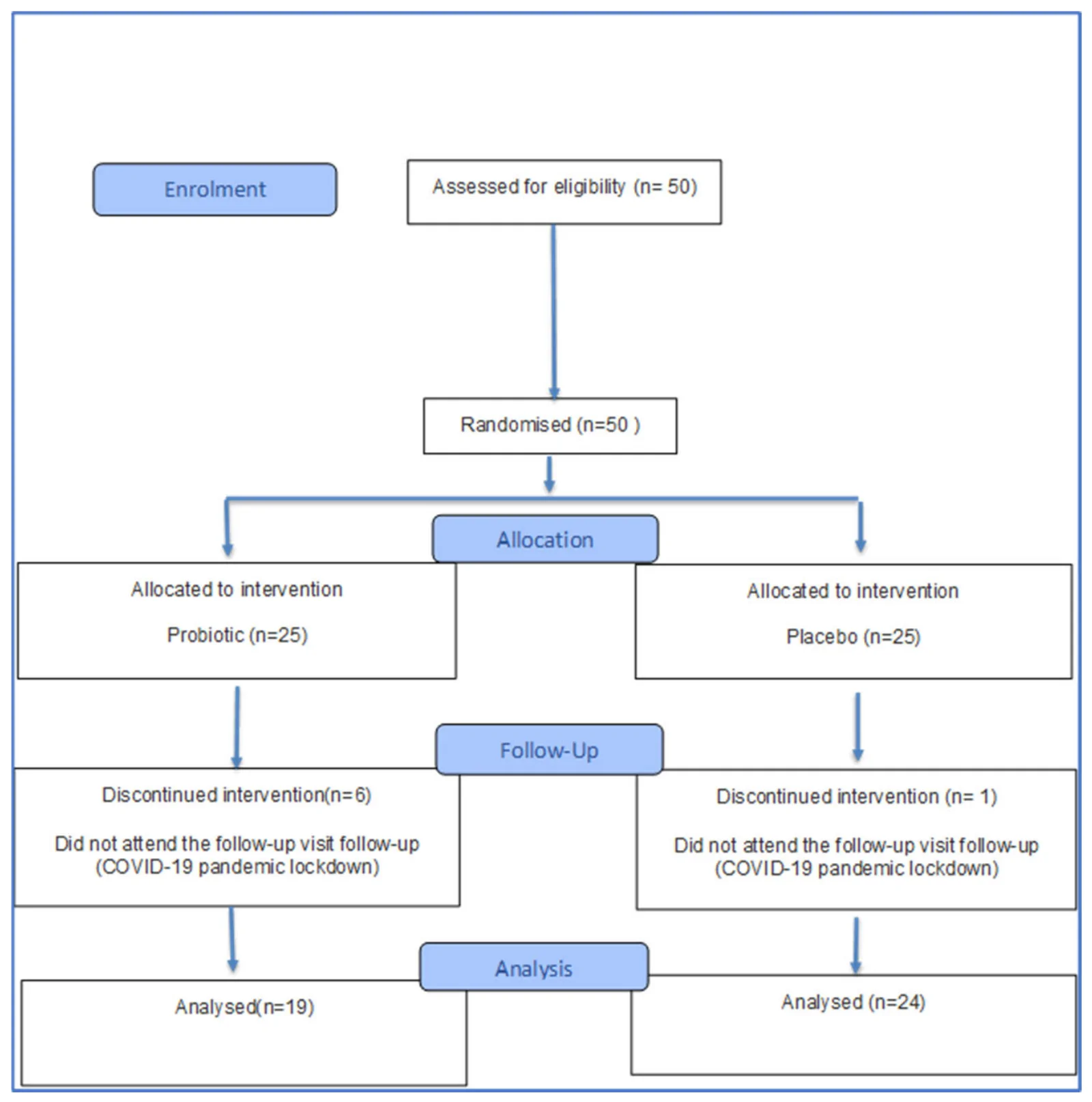
CONSORT flow diagram of participant recruitment, allocation, follow-up, and analysis.

Participants were aged 19–42 years (28.47 ± 5.87) and were randomized to the probiotic (*n* = 25) or placebo group (*n* = 25). No formal a priori sample size calculation was performed for the metabolic outcomes analyzed in this secondary analysis, which were not specified as primary or secondary endpoints in the original trial registration. Therefore, this analysis was considered exploratory and based on the available sample. Participants were randomized 1:1 using a computer-generated sequence prepared in Microsoft Excel by an investigator not involved in recruitment or outcome assessment. Allocation was concealed using sequentially numbered, opaque, sealed envelopes opened after enrollment. Participants and investigators involved in outcome assessment were blinded to treatment allocation, and the probiotic and placebo capsules were identical in appearance. Forty-three participants completed the intervention (probiotic: *n* = 19; placebo: *n* = 24). Seven participants did not complete the post-intervention assessment and were therefore not included in the complete-case analysis; six were from the probiotic group and one from the placebo group. All seven were classified as lost to follow-up because they did not attend the post-intervention assessment. The participant flow through the study is presented in Figure 1.

Participants were randomly assigned to receive either a probiotic preparation or placebo. The probiotic group received SanProbi^®^ Barrier (Sanprobi Sp. z o.o., Szczecin, Poland), a multispecies probiotic formulation containing nine bacterial strains: *Bifidobacterium bifidum* W23, *Bifidobacterium lactis* W52, *Lactobacillus acidophilus* W37, *Lactobacillus brevis* W63, *Lactobacillus casei* W56, *Lactobacillus salivarius* W24, *Lactococcus lactis* W19, *Bifidobacterium lactis* W51, and *Lactococcus lactis* W58. Each capsule contained 5 × 10^8^ colony-forming units (CFU), providing a total daily dose of 1.0 × 10^9^ CFU taken in two divided doses. The placebo consisted of identical-looking capsules containing mainly maize starch and maltodextrins, without probiotic strains. The probiotic and placebo preparations were stored in accordance with the manufacturer’s recommendations throughout the study period. Participants in both groups were instructed to take one capsule twice daily for 12 weeks and to maintain their usual diet and level of physical activity throughout the intervention period. Dietary intake and physical activity were not quantitatively monitored during the intervention. Adherence to the intervention was assessed by participant self-report during follow-up visits. Participants were instructed not to use additional probiotic preparations during the intervention. Adverse events and treatment-related side effects were monitored throughout the follow-up period; none were observed or reported.

### 2.2. Biochemical Tests

Venous blood samples were collected after an overnight fast of at least 8 h at baseline and after the 12-week intervention. Fasting glucose, fasting insulin, and lipid profile parameters were assessed, including total cholesterol, triglycerides, low-density lipoprotein cholesterol (LDL-C), and high-density lipoprotein cholesterol (HDL-C), using an automated biochemical analyzer (Roche Cobas, Roche Diagnostics, Mannheim, Germany) at both time points in the same laboratory. LDL-C was measured directly. For the oral glucose tolerance test (OGTT), participants were administered 75 g of oral glucose, and glucose and insulin concentrations were measured at 120 min after glucose administration. Glucose concentrations were determined using the enzymatic hexokinase method, whereas insulin levels were measured using electrochemiluminescence immunoassay (ECLIA).

### 2.3. Statistical Analysis

Statistical analyses were performed using R software version 4.5.2 (R Foundation for Statistical Computing, Vienna, Austria) and STATISTICA version 13.1 (TIBCO Software Inc., Palo Alto, CA, USA). None of the metabolic outcomes evaluated in the present secondary analysis had been prespecified as primary or secondary endpoints in either the original trial protocol or trial registration. Accordingly, the present metabolic analyses were considered exploratory.

Descriptive statistics were used to summarize the baseline characteristics of participants included in the main complete-case analysis. Continuous variables are presented as mean ± standard deviation (SD) or median and interquartile range (IQR), as appropriate. Baseline balance between the study groups was assessed using standardized mean differences (SMDs), without significance testing of baseline differences.

The distribution of continuous variables was assessed using the Shapiro–Wilk test together with visual inspection of histograms and Q–Q plots. Because of the relatively small group sizes, decisions regarding data presentation and transformation were not based solely on the Shapiro–Wilk *p* value but on the combined assessment of statistical and graphical diagnostics. Variables showing clear positive skewness for which logarithmic transformation improved distributional characteristics and model residual diagnostics were analyzed on the log scale. Accordingly, fasting insulin, 120 min insulin, and HOMA-IR were logarithmically transformed before analysis. Triglycerides showed a positively skewed marginal distribution; however, logarithmic transformation did not improve the residual distribution of the ANCOVA model, and they were therefore analyzed on the original scale. Full Shapiro–Wilk test results and distributional diagnostics are provided in Supplementary Table S1.

The effects of the 12-week probiotic intervention on markers of glucose homeostasis (fasting glucose and insulin, 120 min glucose and insulin during the oral glucose tolerance test [OGTT], and HOMA-IR) and lipid metabolism (total cholesterol, HDL cholesterol, LDL cholesterol, and triglycerides) were evaluated using separate analysis of covariance (ANCOVA) models for each outcome. In each model, the post-intervention value was the dependent variable, the treatment group was included as a fixed factor, and the corresponding baseline value was included as a covariate. The assumption of homogeneity of regression slopes was assessed by testing the interaction between treatment group and the corresponding baseline value. When the interaction was not statistically significant, the final ANCOVA model was fitted without the interaction term. The main ANCOVA analyses were complete-case analyses, including participants with available baseline and post-intervention measurements for the respective outcome.

As a sensitivity analysis, the ANCOVA models were re-estimated using heteroscedasticity-consistent HC3 robust standard errors to assess the robustness of the main findings to potential heteroscedasticity. Results of these analyses are presented in Supplementary Table S2.

In addition, an intention-to-treat (ITT) sensitivity analysis was performed including all 50 randomized participants according to their original treatment allocation. Missing post-intervention measurements were handled using multiple imputation by chained equations with predictive mean matching. Fifty imputed datasets were generated. For each directly imputed outcome, the imputation model included treatment allocation, the corresponding baseline outcome value, age, and baseline BMI as predictors. Only missing post-intervention values were imputed. For fasting insulin and 120 min insulin, imputation was performed on the logarithmic scale, consistent with their subsequent analysis. The ANCOVA model used in the main analysis was fitted separately to each imputed dataset, and parameter estimates and standard errors were combined using Rubin’s rules. For HOMA-IR, missing post-intervention values were derived from the imputed fasting glucose and fasting insulin concentrations rather than imputed independently. Results of the ITT sensitivity analysis are presented in Supplementary Table S3.

Treatment effects were reported as adjusted mean differences (probiotic minus placebo) for outcomes analyzed on the original scale and as geometric mean ratios (GMRs; probiotic/placebo) for outcomes analyzed after logarithmic transformation, together with their 95% confidence intervals (95% CIs) and corresponding two-sided *p* values. Statistical significance was defined as *p* < 0.05. Given the exploratory nature of the analyses and the absence of a prespecified hierarchy among the metabolic outcomes, no adjustment for multiple comparisons was applied. Accordingly, the results were interpreted with emphasis on effect estimates and their 95% CIs rather than on statistical significance alone.

## 3. Results

Of the 50 randomized participants, 43 attended the post-intervention assessment and were available for the main complete-case analyses (placebo, *n* = 24; probiotic, *n* = 19). Seven participants did not attend the post-intervention assessment and were classified as lost to follow-up (one in the placebo group and six in the probiotic group). No participant discontinued the intervention because of an adverse event, and no treatment-related adverse events were reported in either group. The reasons for non-attendance at the follow-up visit are presented in the CONSORT flow diagram. Follow-up measurements were available for 43 participants for most metabolic outcomes. For the 120 min OGTT glucose and insulin measurements, data were available for 39 participants (placebo, *n* = 24; probiotic, *n* = 15) because of additional missing measurements. LDL cholesterol was available for 42 participants (placebo, *n* = 23; probiotic, *n* = 19).

Table 1 summarizes the baseline demographic, anthropometric, and biochemical characteristics of participants included in the main complete-case analysis. Standardized mean differences indicated small-to-moderate baseline imbalances for some variables, particularly in 120 min glucose and triglyceride concentrations.

Adjusted post-intervention outcomes estimated using ANCOVA are presented in Table 2. After adjustment for the corresponding baseline values, no statistically significant between-group differences were observed for fasting glucose, fasting insulin, 120 min glucose, 120 min insulin, HOMA-IR, total cholesterol, HDL cholesterol, LDL cholesterol, or triglycerides. All 95% confidence intervals for the treatment effects included the null value.

Sensitivity analyses using HC3 robust standard errors yielded comparable estimates and did not alter the statistical significance of any treatment effect (Supplementary Table S2). Similarly, the ITT sensitivity analysis based on multiple imputation and including all 50 randomized participants yielded effect estimates and 95% confidence intervals consistent with those obtained in the complete-case analysis, with no statistically significant treatment effects for any metabolic outcome (Supplementary Table S3).

## 4. Discussion

The present findings should be interpreted in the context of the growing recognition of PCOS as a complex metabolic–endocrine disorder rather than solely a reproductive disease [8]. Insulin resistance, hyperinsulinemia, dyslipidemia, and chronic low-grade inflammation are common features of the syndrome and contribute to increased long-term cardiometabolic risk. Therefore, interventions targeting metabolic dysfunction represent an important therapeutic approach in women with PCOS. In recent years, the gut microbiota has emerged as a potential regulator of metabolic homeostasis, providing a rationale for microbiota-targeted strategies such as probiotic supplementation.

Previous randomized controlled trials investigating probiotics and synbiotics in women with PCOS have reported heterogeneous effects on metabolic outcomes [18,19,20,21,22,23,24]. For instance, Zhang et al. reported that probiotic supplementation was associated with improvements in glucose metabolism parameters, including reductions in fasting glucose, insulin concentrations, and HOMA-IR in women with PCOS [18]. Furthermore, Luo et al. investigated the effects of probiotic supplementation in a Chinese population of women with PCOS and reported that the addition of a *Bifidobacterium*-containing probiotic formulation to metformin therapy resulted in a significant reduction in HOMA-IR [19]. Unlike some previous trials, participants in the present study did not receive metformin treatment, and this difference should be considered when interpreting the findings, as metformin is an established insulin-sensitizing agent that may independently improve insulin resistance and lead to reductions in HOMA-IR values [1]. Similarly, Bodake et al. and Darvishi et al. reported significant improvements in fasting glucose and insulin levels, as well as HOMA-IR, following probiotic supplementation in women with PCOS [20,21].

Conversely, Karimi et al. found that 12 weeks of synbiotic supplementation did not significantly affect fasting glucose, insulin levels, or HOMA-IR in women with PCOS [22]. Additionally, Chudzicka-Strugała et al. demonstrated improvements in glycemic parameters in both the intervention and control groups following the intervention period [23]. However, participants in that trial also underwent an intensive lifestyle modification program, including dietary changes and increased physical activity, which may have contributed to the observed metabolic improvements. A similar combination of lifestyle modification and probiotic supplementation was investigated by Kaur et al., who reported improvements in fasting glucose levels, which further highlights the potential contribution of lifestyle-related factors to metabolic outcomes [24].

The heterogeneity of previous studies may be broadly attributed to differences in probiotic formulations, intervention duration, and concomitant interventions. Probiotic formulations varied in bacterial strains and combinations, while intervention periods ranged from 12 weeks to 6 months. In addition, some studies evaluated probiotics as an adjunct to metformin therapy or structured lifestyle modification, whereas others assessed probiotics as a standalone intervention. Differences in baseline metabolic characteristics and study populations may have further contributed to the variability of observed effects. These methodological differences should therefore be considered when comparing metabolic outcomes across trials.

These differences may contribute to the heterogeneous metabolic effects reported across probiotic trials. Probiotic effects are highly strain-specific and may depend on bacterial composition, dosage, duration of supplementation, and interactions with the host microbiome [25,26]. In addition to strain composition, both probiotic dose and intervention duration may influence the magnitude and detectability of metabolic effects. The relatively low daily dose used in the present study (1 × 10^9^ CFU/day) may have been insufficient to induce measurable changes in glucose or lipid metabolism. Similarly, although 12 weeks is a commonly used intervention period in probiotic trials, this duration may not have been sufficient to produce sustained alterations in the gut microbiota and downstream metabolic pathways. Therefore, the absence of significant metabolic effects in the present study may partly reflect the dose and duration of supplementation rather than a complete absence of biological effect. Differences in the metabolic activity of individual probiotic strains, including their ability to modulate inflammation, intestinal permeability, and short-chain fatty acid production, may contribute to variability in their effects on insulin sensitivity and glucose metabolism [27]. Moreover, some previous trials combined probiotic supplementation with additional interventions, such as metformin therapy or intensive lifestyle modification, which may have independently influenced glucose metabolism and HOMA-IR values. In contrast, the present study evaluated probiotic supplementation without concomitant pharmacological treatment or structured lifestyle modification. Therefore, the findings provide an assessment of probiotic supplementation in the absence of concomitant pharmacological or structured lifestyle interventions, although lifestyle factors were not systematically controlled.

Evidence regarding the effects of probiotic supplementation on the lipid profile in women with PCOS remains inconsistent, with studies reporting variable effects on total cholesterol, LDL-C, and HDL-C levels. In a non-PCOS population, Zhang et al. reported that supplementation with *Adlercreutzia equolifaciens* was associated with significant reductions in fasting glucose levels and improvements in lipid metabolism, including decreased cholesterol concentrations [28]. However, the lack of a PCOS-specific population limits the direct comparability of these findings with our study. Conversely, Tabrizi et al. reported that probiotic supplementation improved triglyceride levels; however, no significant effects were observed for total cholesterol, LDL-C, or HDL-C concentrations [29]. Similarly, Samimi et al. reported a significant reduction only in triglyceride concentrations following synbiotic supplementation in women with PCOS, while no significant changes were observed in the remaining lipid parameters [30].

Karamali et al. did not observe significant improvements in metabolic profiles following probiotic supplementation in women with PCOS [31], which is in line with our findings showing no significant changes in lipid parameters after a 12-week intervention. The absence of statistically significant between-group differences should be interpreted with caution, as the relatively small sample size and the corresponding confidence intervals limit the precision with which modest treatment effects can be estimated. For example, the 95% confidence interval for fasting glucose was −0.93 to 6.29 mg/dL, while that for HOMA-IR corresponded to a geometric mean ratio of 0.82–1.26, indicating that the data remain compatible with a relative reduction of approximately 18% as well as an increase of up to approximately 26% in HOMA-IR. Thus, the absence of statistical significance should not be interpreted as evidence of no effect, and larger, prospectively powered randomized trials are needed to determine whether smaller metabolic effects may be present.

The absence of significant changes in lipid parameters observed in our study may be related to variations in probiotic formulations, intervention duration, and study populations compared with previous reports. Importantly, it should be emphasized again that our study evaluated probiotic supplementation without concomitant pharmacological treatment or structured lifestyle modification, which may provide additional insight into the potential metabolic effects of probiotics as a standalone intervention. However, further well-designed studies are required to clarify the potential role of probiotics in modulating lipid metabolism in women with PCOS.

Interestingly, although probiotic supplementation in the same cohort was previously associated with a reduction in BMI from 28.38 to 27.36 kg/m^2^ (−1.02 kg/m^2^; *p* = 0.0198) [15], these findings were not accompanied by significant improvements in glucose homeostasis or lipid profile in the present analysis. The different patterns observed between endocrine and metabolic outcomes in the same cohort may suggest that the effects of probiotic supplementation could differ across clinical domains of PCOS, although this interpretation requires confirmation in larger studies.

One possible explanation for the dissociation between endocrine and metabolic outcomes is that probiotic effects may involve partially distinct pathways within the gut–ovary and gut–metabolic axes. Beyond their established metabolic and immunomodulatory effects, microbiota-derived metabolites may influence ovarian endocrine function through pathways involving steroid metabolism and bile acid signaling [7,11,12]. Conversely, effects on systemic insulin sensitivity and lipid metabolism may require more pronounced or sustained changes in microbial composition and metabolite production. These pathways are interconnected but may not respond uniformly to the same probiotic formulation, dose, or duration of supplementation. Thus, the hormonal and BMI changes previously observed in this cohort may reflect effects on gut–ovary signaling that are not necessarily accompanied by measurable improvements in systemic glucose or lipid metabolism over a 12-week period. However, as microbial composition and metabolite profiles were not assessed in the present study, these mechanisms remain hypothetical and require confirmation in studies incorporating microbiome and metabolomic analyses.

As illustrated by the studies discussed above, evidence regarding the metabolic effects of probiotic supplementation in women with PCOS remains limited and heterogeneous. Moreover, data from Central European populations, particularly Polish women with PCOS, are scarce. Therefore, the present study may contribute to filling this gap by providing evidence from a well-characterized Polish cohort.

### Strengths and Limitations

The strengths of the present study include its randomized, double-blind, placebo-controlled design, which minimizes the risk of selection and observer bias. Furthermore, baseline values were taken into account using analysis of covariance, an approach that increases statistical efficiency and provides unbiased estimates of treatment effects in randomized trials. In addition, sensitivity analyses using heteroscedasticity-consistent HC3 robust standard errors confirmed the robustness of the main findings. The robustness of the findings was further supported by an ITT sensitivity analysis using multiple imputation, which yielded treatment-effect estimates consistent with the complete-case analyses. To our knowledge, this is among the few studies evaluating the metabolic effects of probiotic supplementation in Polish women with PCOS.

Several limitations should also be acknowledged. First, the relatively small sample size may have limited the statistical power to detect modest treatment effects. In addition, no formal a priori power calculation was performed for the metabolic outcomes, resulting in relatively wide confidence intervals around the treatment effect estimates. Consequently, the present secondary analysis was considered exploratory, and modest treatment effects cannot be completely excluded. Confidence intervals were particularly wide for the 120 min OGTT outcomes and triglycerides; therefore, the absence of statistically significant differences should not be interpreted as evidence of equivalence or the absence of a metabolic effect. The analysis included only participants with available post-intervention assessments, and differential attrition (6 participants in the probiotic group versus 1 in the placebo group) may have introduced attrition bias. Although the ITT sensitivity analysis yielded findings consistent with the complete-case analyses, bias related to missing follow-up data cannot be entirely excluded. Second, although participants were instructed to maintain their habitual diet and level of physical activity throughout the intervention, these factors were not objectively quantified, and changes in lifestyle or exposure to dietary sources of probiotics cannot therefore be completely excluded. Antibiotic use, prebiotic supplementation, and consumption of fermented foods were not systematically monitored during the intervention and may have contributed to variability in treatment response. Adherence was assessed by self-report rather than by objective measures, which may have introduced additional variability in treatment exposure. The absence of phenotype-specific analyses also represents a limitation of the present study. Categorical glucose abnormalities, such as impaired fasting glucose and impaired glucose tolerance, were not specifically analyzed. The 12-week intervention period and relatively low probiotic dose (1 × 10^9^ CFU/day) do not allow conclusions regarding the long-term sustainability of the observed effects, and the findings should therefore not be extrapolated to longer-term probiotic use. Therefore, larger randomized controlled trials with adequate statistical power, longer intervention periods, and comprehensive assessment of lifestyle factors are warranted to confirm these findings. The findings are specific to the multispecies probiotic formulation used in this study and should not be generalized to other probiotic strains or supplementation protocols without further evidence. Finally, the metabolic outcomes examined in this secondary analysis were not specified among the primary or secondary outcome measures in the ClinicalTrials.gov registration. Although these parameters were prospectively collected as part of the original study procedures, the absence of prospective registration of the specific metabolic outcomes should be acknowledged as a limitation. Accordingly, interpretation should emphasize the magnitude and precision of treatment-effect estimates rather than statistical significance alone.

## 5. Conclusions

In conclusion, multispecies probiotic supplementation for 12 weeks was not associated with significant improvements in glucose metabolism, insulin resistance, or lipid profile in women with PCOS compared with placebo. These findings contribute to the growing yet heterogeneous evidence regarding the metabolic effects of probiotics in PCOS and highlight the need for larger, adequately powered randomized controlled trials with longer follow-up periods.

Notably, although probiotic supplementation was previously associated with improvements in hormonal parameters and BMI in this cohort, similar effects were not observed for metabolic outcomes in the present analysis [15]. This observation warrants further investigation into the relationship between hormonal and metabolic responses to probiotic supplementation in women with PCOS.

## Figures and Tables

**Table 1:** Baseline characteristics of participants included in the main complete-case analysis.

Variable	Placebo (*n* = 24)	Probiotic (*n* = 19)	SMD
Age (years)	28.38 ± 5.38	28.47 ± 5.87	0.0176
Body weight (kg)	80.54 ± 15.13	76.19 ± 17.83	−0.2268
BMI (kg/m^2^)	28.67 ± 5.45	28.47 ± 5.59	−0.0363
Fasting glucose (mg/dL)	87.72 ± 17.40	85.89 ± 9.41	−0.2428
120 min glucose (mg/dL)	104.48 ± 23.86	113.53 ± 27.45	0.3546
Fasting insulin (µIU/mL)	10.95 (7.20; 13.18)	13.00 (7.35; 16.20)	0.2653
120 min insulin (µIU/mL)	49.50 (34.80; 87.05)	81.10 (46.85; 118.30)	0.2316
HOMA-IR	2.32 (1.52; 2.87)	2.89 (1.55; 3.73)	0.2195
Total cholesterol (mg/dL)	187.01 ± 30.24	191.73 ± 29.29	0.1580
HDL cholesterol (mg/dL)	57.88 ± 11.27	59.58 ± 16.56	0.1227
LDL cholesterol (mg/dL)	114.58 ± 26.54	114.63 ± 35.24	0.0017
Triglycerides (mg/dL)	78.00 (63.78; 132.50)	97.80 (47.90; 177.90)	0.3695

Data are presented as mean ± SD for approximately normally distributed variables and as median (IQR) for variables with skewed distributions. SMD, standardized mean difference.

**Table 2:** Adjusted post-intervention estimates and treatment effects estimated using ANCOVA.

Outcome	Adjusted Placebo Mean (95% CI)	Adjusted Probiotic Mean (95% CI)	Adjusted Effect (95% CI)	*p* Value
Fasting glucose (mg/dL)	86.33 (83.94–88.72)	89.01 (86.32–91.70)	2.68 (−0.93 to 6.29)	0.141
Fasting insulin (µIU/mL)	10.60 (9.38–11.96)	10.38 (9.06–11.91)	0.98 (0.82–1.18) *	0.826
120 min glucose (mg/dL)	106.55 (97.08–116.02)	112.97 (100.93–125.00)	6.42 (−9.05 to 21.89)	0.406
120 min insulin (µIU/mL)	48.40 (35.55–65.88)	54.07 (36.55–79.99)	1.12 (0.68–1.85) *	0.657
HOMA-IR	2.25 (1.95–2.59)	2.28 (1.94–2.67)	1.01 (0.82–1.26) *	0.894
Total cholesterol (mg/dL)	180.94 (173.46–188.43)	184.41 (175.99–192.82)	3.46 (−7.81 to 14.74)	0.538
HDL cholesterol (mg/dL)	59.84 (56.77–62.90)	61.74 (58.29–65.19)	1.91 (−2.71 to 6.53)	0.409
LDL cholesterol (mg/dL)	106.08 (99.59–112.56)	105.55 (98.41–112.68)	−0.53 (−10.17 to 9.11)	0.912
Triglycerides (mg/dL)	107.98 (94.26–121.71)	110.87 (95.41–126.32)	2.88 (−17.96 to 23.72)	0.781

* Adjusted group estimates are presented as adjusted means for outcomes analyzed on the original scale and as back-transformed geometric means for log-transformed outcomes. Treatment effects are presented as adjusted mean differences (probiotic minus placebo) for outcomes analyzed on the original scale and as geometric mean ratios (probiotic/placebo) for log-transformed outcomes (fasting insulin, 120 min insulin, and HOMA-IR).

## Data Availability

The data presented in this study are available upon request from the corresponding author.

## Supplementary Materials

The following supporting information can be downloaded at https://www.mdpi.com/article/10.3390/nu18183090/s1, Table S1: Shapiro–Wilk tests and distributional diagnostics supporting data presentation and transformation decisions; Table S2: Sensitivity analysis of treatment effects using ANCOVA with HC3 heteroscedasticity-consistent robust standard errors; Table S3: Comparison of complete-case and intention-to-treat multiple-imputation ANCOVA estimates for metabolic outcomes.
