# Supplementary material for: More than Hormones? Metabolic Effects of Twelve-Week Probiotic Supplementation in Women with Polycystic Ovary Syndrome: A Secondary Analysis of a Randomized Double-Blind Placebo-Controlled Study
Source: Nutrients. 2026 Sep 21;18(18):3090. doi: 10.3390/nu18183090 (PMC13610117; doi:10.3390/nu18183090)
Supplement: Supplementary file 1 [file nutrients-18-03090-s001.zip › nutrients-4495460-supplementary.pdf]

Table S1. Shapiro–Wilk tests and distributional diagnostics supporting data presentation and transformation decisions

Panel A. Shapiro–Wilk tests for observed metabolic variables on the original scale

Outcome	Baseline placebo W (p)	Baseline probiotic W (p)	Post placebo W (p)	Post probiotic W (p)	Final ANCOVA scale
Fasting glucose	0.978 (0.865)	0.961 (0.593)	0.965 (0.552)	0.964 (0.645)	Original
Fasting insulin	0.887 (0.012)	0.924 (0.135)	0.863 (0.004)	0.832 (0.003)	Log
120-min glucose	0.949 (0.255)	0.959 (0.550)	0.932 (0.106)	0.958 (0.660)	Original
120-min insulin	0.760 (<0.001)	0.835 (0.004)	0.747 (<0.001)	0.949 (0.516)	Log
HOMA-IR	0.860 (0.003)	0.876 (0.018)	0.826 (<0.001)	0.804 (0.001)	Log
Total cholesterol	0.971 (0.683)	0.947 (0.356)	0.984 (0.956)	0.884 (0.025)	Original
HDL cholesterol	0.961 (0.451)	0.938 (0.248)	0.970 (0.669)	0.870 (0.015)	Original
LDL cholesterol	0.913 (0.040)	0.990 (0.999)	0.921 (0.069)	0.924 (0.135)	Original
Triglycerides	0.910 (0.036)	0.894 (0.038)	0.904 (0.027)	0.890 (0.032)	Original

Sample sizes: baseline values were evaluated among participants who completed the intervention: placebo, n = 24; probiotic, n = 19. Post-intervention sample sizes were placebo n = 24 and probiotic n = 19, except for 120-min glucose and 120-min insulin (probiotic, n = 15) and LDL cholesterol (placebo, n = 23).

Panel B. Shapiro–Wilk tests of ANCOVA residuals for variables considered for logarithmic transformation

Outcome	N	Original-scale residuals W (p)	Log-scale residuals W (p)	Final ANCOVA scale
Fasting insulin	43	0.847 (<0.001)	0.941 (0.029)	Log
120-min insulin	39	0.836 (<0.001)	0.977 (0.590)	Log
HOMA-IR	43	0.851 (<0.001)	0.947 (0.048)	Log
Triglycerides	43	0.939 (0.023)	0.921 (0.006)	Original

W, Shapiro–Wilk test statistic. Distributional assessment was based on the Shapiro–Wilk test together with visual inspection of histograms and Q–Q plots. Transformation decisions were not based solely on a Shapiro–Wilk p-value threshold but also considered the direction and magnitude of skewness and the distribution of ANCOVA residuals. Fasting insulin, 120-min insulin, and HOMA-IR showed marked positive skewness, and logarithmic transformation improved distributional and residual diagnostics; these outcomes were therefore analyzed on the log scale. Although triglycerides showed positive skewness on the original scale, logarithmic transformation did not improve the ANCOVA residual distribution and triglycerides were therefore retained on the original scale. Isolated Shapiro–Wilk p values < 0.05 in other variables did not automatically result in transformation in the absence of marked positive skewness or consistent graphical evidence of problematic distributional departure.

Table S2. Sensitivity analysis of treatment effects using ANCOVA with HC3 heteroscedasticity-consistent robust standard errors

**Outcome**	**N**	**Adjusted effect (95% CI), HC3**	**p value**
Fasting glucose (mg/dL)	43	2.68 (−1.11 to 6.47)	0.161
Fasting insulin (μIU/mL)	43	0.98 (0.81–1.19)*	0.836
120-min glucose (mg/dL)	39	6.42 (−11.61 to 24.44)	0.475
120-min insulin (μIU/mL)	39	1.12 (0.64–1.94)*	0.687
HOMA-IR	43	1.01 (0.81–1.27)*	0.901
Total cholesterol (mg/dL)	43	3.46 (−8.45 to 15.37)	0.560
HDL cholesterol (mg/dL)	43	1.91 (−3.24 to 7.05)	0.458
LDL cholesterol (mg/dL)	42	−0.53 (−10.70 to 9.64)	0.917
Triglycerides (mg/dL)	43	2.88 (−17.49 to 23.26)	0.776

Treatment effects were estimated using ANCOVA with the post-intervention value as the dependent variable, treatment group as the fixed factor, and the corresponding baseline value as a covariate. Heteroscedasticity-consistent HC3 robust standard errors were used to calculate 95% confidence intervals and p values. Effects are presented as adjusted mean differences (probiotic minus placebo) for outcomes analyzed on the original scale and as geometric mean ratios (probiotic/placebo) for log-transformed outcomes (*fasting insulin, 120-min insulin, and HOMA-IR).

Table S3. Comparison of complete-case and intention-to-treat multiple-imputation ANCOVA estimates for metabolic outcomes.

Outcome	Complete-case N	Complete-case adjusted effect (95% CI)	p value	ITT N	ITT-MI adjusted effect (95% CI)	p value
Fasting glucose (mg/dL)	43	2.68 (−0.93 to 6.29)	0.141	50	2.50 (−0.95 to 5.94)	0.150
Fasting insulin (μIU/mL)	43	0.98 (0.82–1.18)*	0.826	50	0.99 (0.82–1.19)*	0.905
120-min glucose (mg/dL)	39	6.42 (−9.05 to 21.89)	0.406	50	7.04 (−9.03 to 23.11)	0.379
120-min insulin (μIU/mL)	39	1.12 (0.68–1.85)*	0.657	50	1.14 (0.70–1.85)*	0.590
HOMA-IR	43	1.01 (0.82–1.26)*	0.894	50	1.02 (0.83–1.25)*	0.868
Total cholesterol (mg/dL)	43	3.46 (−7.81 to 14.74)	0.538	50	3.85 (−7.00 to 14.70)	0.477
HDL cholesterol (mg/dL)	43	1.91 (−2.71 to 6.53)	0.409	50	1.96 (−2.70 to 6.62)	0.400
LDL cholesterol (mg/dL)	42	−0.53 (−10.17 to 9.11)	0.912	50	0.28 (−9.03 to 9.59)	0.951
Triglycerides (mg/dL)	43	2.88 (−17.96 to 23.72)	0.781	50	1.31 (−19.98 to 22.60)	0.901

Complete-case estimates were obtained from ANCOVA models including participants with available baseline and post-intervention measurements for the respective outcome. The intention-to-treat (ITT) analysis included all 50 randomized participants according to their original treatment allocation. Missing post-intervention measurements were handled using multiple imputation by chained equations with predictive mean matching (50 imputations), and estimates were combined using Rubin's rules. Effects are presented as adjusted mean differences (probiotic minus placebo) for outcomes analyzed on the original scale and as geometric mean ratios (probiotic/placebo) for log-transformed outcomes (*fasting insulin, 120-min insulin, and HOMA-IR). For HOMA-IR, missing post-intervention values were derived from the imputed fasting glucose and fasting insulin concentrations rather than imputed independently.
